# Effects of Preparation Conditions on the Efficiency of Visible-Light-Driven Hydrogen Generation Based on Ni(II)-Modified Cd_0_._25_Zn_0_._75_S Photocatalysts

**DOI:** 10.3390/molecules27134296

**Published:** 2022-07-04

**Authors:** Maali-Amel Mersel, Lajos Fodor, Péter Pekker, Éva Makó, Ottó Horváth

**Affiliations:** 1 Research Group of Environmental and Inorganic Photochemistry, Center for Natural Sciences, Faculty of Engineering, University of Pannonia, P.O. Box 1158, H-8210 Veszprém, Hungary; sam003miloo@gmail.com (M.-A.M.); lajos@almos.uni-pannon.hu (L.F.); 2 Environmental Mineralogy Research Group, Research Institute of Biomolecular and Chemical Engineering, University of Pannonia, P.O. Box 1158, H-8210 Veszprem, Hungary; pekkerpeter@gmail.com; 3 Department of Materials Engineering, Research Center for Engineering Sciences, University of Pannonia, P.O. Box 1158, H-8210 Veszprem, Hungary; makoe@almos.uni-pannon.hu

**Keywords:** photocatalysis, hydrogen generation, visible-light-driven, solar energy conversion, ZnS-CdS composite, NiS, preparation conditions

## Abstract

Hydrogen as an environmentally friendly fuel can be produced by photocatalytic procedures from aqueous systems, utilizing H_2_S, an industrial side-product, by conversion and storage of renewable solar energy. Although composites of CdS and ZnS prepared by co-precipitation are very efficient in heterogeneous photocatalytic H_2_ generation, the optimal conditions for their synthesis and the effects of the various influencing factors are still not fully clarified. In this work, we investigated how the efficiency of Cd_0_._25_Zn_0_._75_S composites modified with Ni(II) was affected by the doping method, Ni-content, hydrothermal treatment, and presence of a complexing agent (ammonia) used in the preparation. The composition, optical, and structural properties of the photocatalysts prepared were determined by ICP, DRS, XRD, TEM, and STEM-EDS. Although hydrothermal treatment proved preferable for Ni-free composites, Ni-modification was more efficient for untreated composites precipitated from ammonia-containing media. The best efficiency (14.9% quantum yield at 380 nm irradiation, 109.8 mmol/g/h hydrogen evolution rate) achieved by surface modification with 0.1–0.3% Ni(II) was 15% and 20% better than those for hydrothermally treated catalyst and similarly prepared Pt-modified one, respectively. Structural characterization of the composites clearly confirmed that the Ni^2+^ ions were not embedded into the CdS-ZnS crystal lattice but were enriched on the surface of particles of the original catalyst in the form of NiO or Ni(OH)_2_. This co-catalyst increased the efficiency by electron-trapping, but its too high amount caused an opposite effect by diminishing the excitable surface of the CdS-ZnS particles.

## 1. Introduction

The refinery of crude oil and the purification of natural gas produce a huge amount of toxic H_2_S gas [1]. Industrial processing is currently carried out by the Claus process producing sulfur and water, wasting hydrogen as a potential energy source [2,3,4]. In contrast, heterogeneous photocatalytic H_2_S splitting yields hydrogen gas instead of water [5,6,7]. In the 21st century, the research activities on water splitting [8,9,10,11] or other heterogeneous photocatalytic hydrogen evolution processes have been significantly accelerated [12,13,14,15]. Most of these are focused on sulfide-type semiconductors, using mainly Na_2_S solution or lactic acid as a sacrificial agent [16,17]. The most efficient catalysts for hydrogen production are based on CdS [8,12,13,15,18,19]. Although CdS can be excited by visible light, the energy level of its conduction band (CB) is not high enough for H_2_ production in alkaline media. To improve this, co-precipitation of CdS with other metal sulfides of high BG, such as MnS [20,21,22] or ZnS [23,24,25,26,27,28,29], is a commonly used method. In this way, the band gap (BG) value is only slightly increased compared to that of CdS, but the CB potential is shifted in the negative direction, making the reduction of water thermodynamically favored. Several research groups have also used the co-precipitation of CdS with NiS of lower BG, but the resulting catalysts have mostly achieved high quantum yield (QY) only in acidic or neutral media with the degradation of lactic acid [30,31,32,33,34,35] or ethanol [36]. He and Guo were the only ones to measure an outstandingly high QY of 74.6% in alkaline media with a hydrothermally treated composite prepared by co-precipitation of CdS and NiS (5% (m/m)) [37].

Studies over the past 10 years have clearly shown that the most efficient catalysts in alkaline media were obtained by co-precipitation of CdS and ZnS. Among these works, the best activity has been reported by those who applied hydrothermal treatment (HTT) to Cd_x_Zn_1–x_S semiconductors [28,31,38,39,40,41]. This positive effect has also been confirmed in our previous work [42]. For these composites, the role of twin boundaries formed during the thermal treatment has been highlighted as the main reason for the good activity [39,41,42,43]. These stacking faults result in the formation of wurtzite–zinc blende heterojunctions that promote the separation of the photogenerated charge carriers, reducing the recombination rate and leading to an increase in photoactivity [37,39,40]. Although the Cd_x_Zn_1–x_S semiconductors of different compositions are highly efficient hydrogen-generating photocatalysts even without modification, further increases in their activity have been reported, using noble metal [44,45,46,47,48,49] or other transition metal-based co-catalysts [15,19,50] or other non-metallic additives such as graphene oxide [15,32,43] or graphitic C_3_N_4_ [31,51]. Several research groups have worked on the substitution of expensive noble metal co-catalysts with low-cost, mainly Ni-containing materials such as Ni [20,31], NiS [38,39,50], or NiO [44]. Wang et al. have modified the surface of Cd_0_._4_Zn_0_._6_S with photochemically deposited CuS, CoS, and NiS. All of these co-catalysts resulted in a 4–5-fold increase in efficiency, although only a 1.2 mmol/g/h H_2_ evolution rate was achieved for the best NiS [50]. Stroyuk et al. also obtained a nearly 5% increase in the quantum yield with application of a high-pressure Hg vapor lamp (310–370 nm) for Cd_0_._5_Zn_0_._5_S, on the surface of which 2% Ni was deposited [20]. A 2.8–3.5-fold increase was achieved by NiS deposition via chemical precipitation on the surface of a hydrothermally treated semiconductor of similar composition [38,39].

In most of these works, either expensive noble metals or energy-consuming heat treatments were used during their synthesis. In this work, we report the development of a hydrogen-generating photocatalyst that is active in the visible-light range and neither contains noble metals nor requires energy-intensive thermal treatment.

## 2. Results and Discussion

### 2.1. Photocatalytic H_2_ Production

#### 2.1.1. Ni(II) and Pt Modified Catalysts

**Figure 1:**
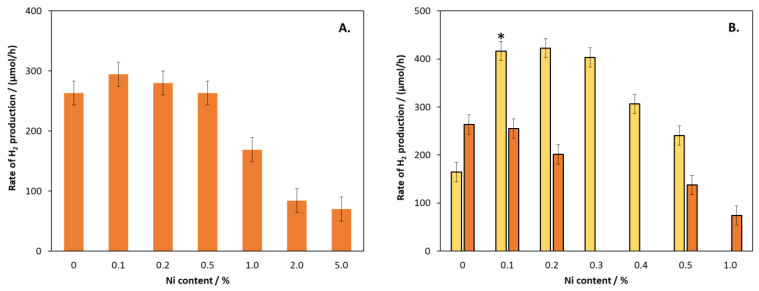
The rate of H_2_ production for catalysts prepared from 2-fold excess of ammonia and modified with different amounts of Ni(II) in the bulk (**A**) and on the surface (**B**). Orange and yellow bars represent the hydrothermally treated and untreated catalysts, respectively. The asterix indicates the catalyst for which the quantum yield has been determined. The primary volume vs. time functions and their time derivatives (as rate of H_2_ evolution) are given in the Supplementary Materials as Figure S1.

The Cd_0_._25_Zn_0_._75_S catalysts were modified by Ni(II) in the bulk (Figure 1A) and on the surface (Figure 1B). For the bulk-modified composites, only a slight increase (about 10%) could be observed at low Ni content, which decreased dramatically above 0.5%. For the surface-modified semiconductors, a considerable difference was observed between the hydrothermally treated and untreated composites, as indicated by the orange and yellow bars in Figure 1B, respectively. The former did not show any increase in efficiency even at low Ni content, while the untreated catalysts resulted in a more than two-fold increase in the rate of H_2_ production (RHP) in the range of 0.1–0.3%. However, at higher Ni contents, a decrease was observed.

For one of the most efficient catalysts (marked with an asterisk in Figure 1B), the quantum yield was determined by using a 380 nm LED as light source. The average RHP was measured to be 1976 μmol H_2_/h for a catalyst mass of 18 mg. Using these data, the quantum yield was calculated to be 14.9%, and the RHP related to the mass of the catalyst was 109.8 mmol/g/h, which is one of the best published values for noble metal-free catalyst. It should be noted that RHP is not a suitable parameter for comparing catalysts because its value depends not only on the quality of the catalyst but also on its amount, the light source, and the composition of the solution phase of the system.

A reasonable explanation for these changes is that Ni(II), either in the bulk or on the surface, promotes the trapping of the CB electrons, but if it covers the surface too much, it inhibits the absorption of photons. The particle size of the hydrothermally untreated sample was smaller, as shown in our previous work [42], so its specific surface area is higher; therefore, the surface is saturated only at higher Ni content.

**Figure 2:**
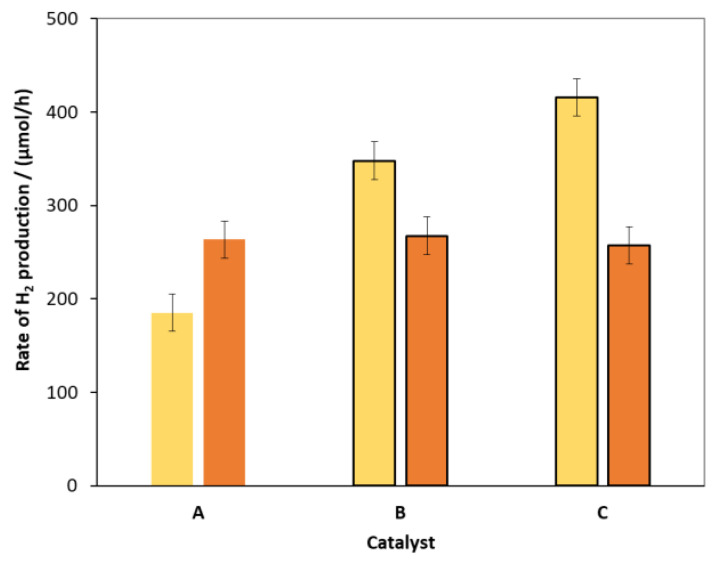
The RHP for unmodified catalysts (**A**) and for catalysts modified with 0.1% Pt (**B**) and 0.1% Ni(II) (**C**) on the surface. Orange and yellow bars represent the hydrothermally treated and untreated catalysts, respectively. The primary volume vs. time functions and their time derivatives (as rate of H_2_ evolution) are given in the Supplementary Materials as Figure S2.

The changes in H_2_ evolution efficiencies of hydrothermally treated and untreated ZnS-CdS catalysts after surface modification with Pt and Ni were compared. As shown in Figure 2, there is no noticeable change for the HT-treated composite. In contrast, for the catalyst not treated hydrothermally, surface modification has significantly increased the catalytic activity in both cases. Compared to the unmodified catalyst, Pt nearly doubled the RHP, while Ni(II) increased it by an additional 20%. These results confirm the advantage of this less expensive and earth-abundant metal over costly noble metals such as Pt or Pd.

#### 2.1.2. Effects of Ammonia as Complexing Agent

**Figure 3:**
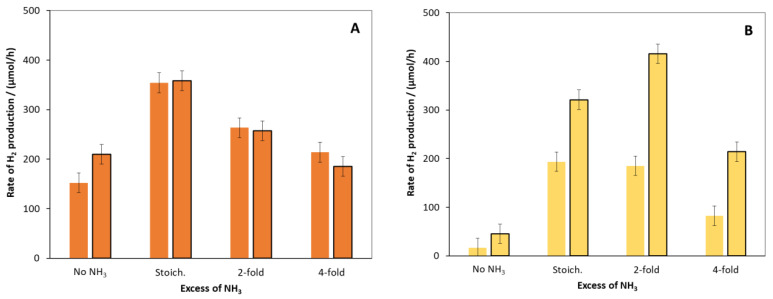
The rate of H_2_ production for catalysts precipitated from solutions containing different amounts of ammonia. (For comparison, the rates for the catalyst prepared without NH_3_, designated as “No NH_3_”, are also shown, along with those for that prepared in the presence of stoichiometric amount of NH_3_, designated as “Stoich.”). (**A**,**B**) show the hydrothermally treated and untreated catalysts, respectively. The bordered bars symbolize the surface modification of catalysts with 0.1% Ni content. The primary volume vs. time functions and their time derivates (as rate of H_2_ evolution) are given in the Supplementary Materials as Figure S3.

Figure 3 shows the effect of the amount of NH_3_ applied during the precipitation. In the case of the hydrothermally treated composites, the surface modification did not increase the catalytic activity except for the least active catalyst, which was prepared without the addition of ammonia. In contrast, 0.1% surface modification significantly increased the catalytic efficiency for all hydrothermally untreated composites. The RHP value of the sample obtained at two-fold ammonia excess proved to be the most active of all the catalysts investigated. Higher ammonia concentration resulted in a decrease in efficiency in all cases. Hence, the catalyst samples for further studies were prepared by using two-fold ammonia excess. The designation of this condition in the sample codes is “2N”.

### 2.2. Characterization of Catalysts

By measuring diffuse reflectance, using the Tauc method, the BG values of bulk-modified and hydrothermally untreated surface-modified composites were determined (Table 1). As in our previous work [42], we found that the BG value of unmodified catalysts slightly decreases (by about 0.1 eV) with heat treatment. The resulting BG essentially did not change by modification with Ni^2+^, indicating that there was no significant chemical interaction between the Ni(II) ions and the CdS-ZnS composite. This was in accordance with previous observations. In the case of a catalyst with a composition (Cd_0_._5_Zn_0_._5_S) relatively similar to that of our samples but prepared in a different way, it was observed that the formation of 0.5% NiS did not modify the absorption edge [50]. For catalysts of Cd_0_._5_Zn_0_._5_S composition, modification with 0.25% Ni caused a BG change from 2.36 to 2.32 eV [39], while in a similar case, no change of the 2.62-eV BG was observed [38]. Modification of pure CdS with 1–9% Ni led to the decrease in the BG from 2.38 to 2.36 eV [37]. The decrease in BG caused by HTT results in the catalyst absorbing about 5–10% more photons, which would justify only a 5–10% increase in the catalytic efficiency. Since the measured RHP indicated a much larger change in the opposite direction to that expected from the change in BG, it is clear that the increase in the activity is not only due to the change in the number of photons absorbed.

**Figure 4:**
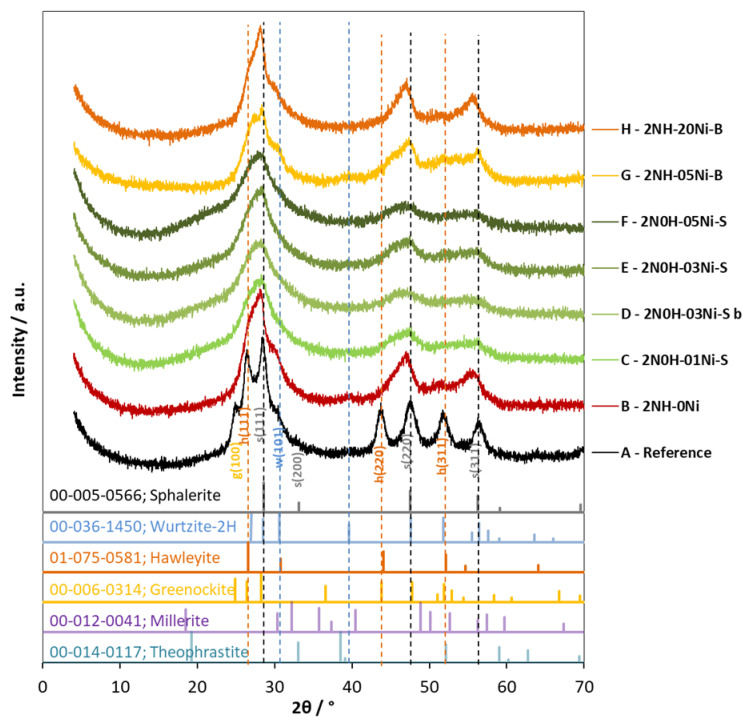
XRD patterns of photocatalysts prepared in the presence of 2-fold excess of ammonia (“2N”). (**A**) Mixture of ZnS and CdS (in 3:1 molar ratio) synthesized and hydrothermally treated (“H”) in the same way as other catalysts. (**B**) Hydrothermally treated unmodified and (**G**) and (**H**) hydrothermally treated and modified with 0.5% and 2.0% Ni(II) in the bulk, respectively. (**C**–**F**) Hydrothermally not treated (“0H”) and modified with 0.1%, 0.3%, 0.3%, and 0.5% Ni(II) on the surface, respectively. (**D**) was measured before (“b”) illumination, while all others were measured after illumination. (For the sake of unambiguity, besides the designation letter, also a more detailed code indicating the preparation conditions is also given for each pattern) The dashed vertical lines designate the characteristic peaks of cubic sphalerite (black), hawleyite (orange), and hexagonal wurtzite (blue).

The catalysts were also characterized by X-ray diffraction (XRD) measurements. The samples were prepared by drying the suspensions of the catalysts (Figure 4). A simple mixture in a 3:1 molar ratio of freshly prepared and hydrothermally treated ZnS and CdS were used as references. From the XRD patterns, it was clearly shown that all the ZnS-CdS samples mainly consisted of cubic sphalerite and hawleyite. Especially the hydrothermally treated samples show a slight shoulder around 30.5° 2θ, indicating the presence of hexagonal wurtzite. The presence of NiS (millerite) or Ni(OH)_2_ (theophrastite) cannot be clearly detected even at 2% of Ni(II) content. This may be caused by the low Ni content and the overlap of the peaks of these compounds with the broadened peaks of sphalerite and hawleyite. The position of the peaks, mainly the ones belonging to CdS, shifted towards higher 2θ, while those belonging to sphalerite slightly moved towards lower 2θ. This suggests that the Zn^2+^ ions are partly substituted by Cd^2+^ in the sphalerite lattice, and the Cd^2+^ ions are slightly replaced by Zn^2+^ in the hawleyite lattice. 

The crystallite size (CS) was also calculated from the full width at half maximum (FWHM) of the 111 reflections of sphalerite (at around 28.559° 2θ), according to the Scherrer equation. Hydrothermally treated catalysts showed about twice as much CS (60 ± 10 Å) as non-hydrothermally treated catalysts (27 ± 8 Å). Modification with Ni, either in the bulk (Figure 4G–H) or on the surface (Figure 4C–F), didn’t cause any shift in the location of the peak maxima or any change in the CS, suggesting that Ni modification does not affect the crystal structure of the unmodified ZnS-CdS composite. XRD patterns of a catalyst modified with 0.3% Ni(II) on the surface obtained before and after usage were compared. XRD patterns D–E in Figure 4 clearly show that three consecutive illuminations did not cause any noticeable change, confirming that there was no significant structural change in the composite during usage.

**Figure 5:**
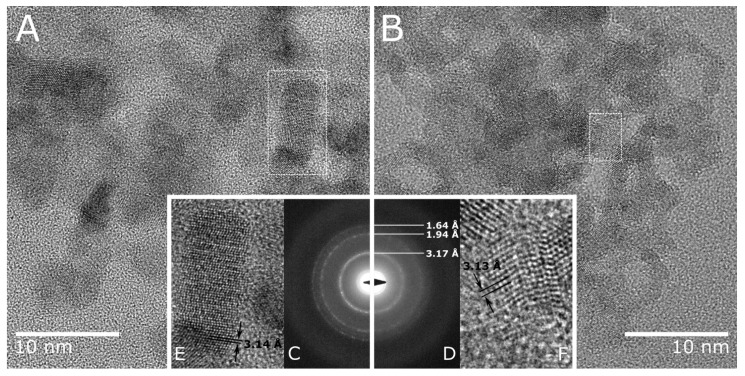
HRTEM images (**A**,**B**), selected area diffraction (SAED) ring patterns (**C**,**D**), and lattice fringes corresponds to the (111) lattice plane (**E**,**F**) of Cat-4H (**A**,**C**,**E**) and Cat-4E (**B**,**D**,**F**).

To better understand the structure of the catalysts, TEM images of a hydrothermally untreated surface-modified composite (Cat-4E, marked with E in Figure 4) and a hydrothermally treated bulk modified composite (Cat-4H, marked with H in Figure 4) were compared. The HRTEM images (Figure 5) show that both composites consist of nearly isometric or slightly elongated particles. The crystal size of the hydrothermally treated Cat-4H is clearly larger than that of Cat-4E, which is consistent with our previously published result [42] that the average crystal size of the Ni-free hydrothermally treated and untreated samples were found to be 12 and 5 nm, respectively. Also similar to the unmodified sample is the presence of stacking faults such as twin boundaries and planar defects.

The crystal size difference is also recognizable in the electron diffraction patterns (Figure 5C,D), with slightly broader diffraction rings produced by the Cat-4E sample and sharper rings by the Cat-4H catalyst, indicating smaller and larger average crystal sizes, respectively. These results are in agreement with the broader peaks of the Cat-4E catalyst in the XRD pattern. The diffraction rings of both Ni-modified samples occur at the same d-values, which are almost identical to the values obtained earlier for unmodified composites (3.17 Å (111), 1.92 Å (220), and 1.64 Å (311)) [42]. The lattice fringes corresponding to the (111) lattice plane were also measured to be similar for Cat-4H (Figure 5E, 3.14 Å) and Cat-4E (Figure 5F, 3.13 Å). These results confirm our previous conclusion that Ni modification does not affect the crystal structure of CdS-ZnS.

**Figure 6:**
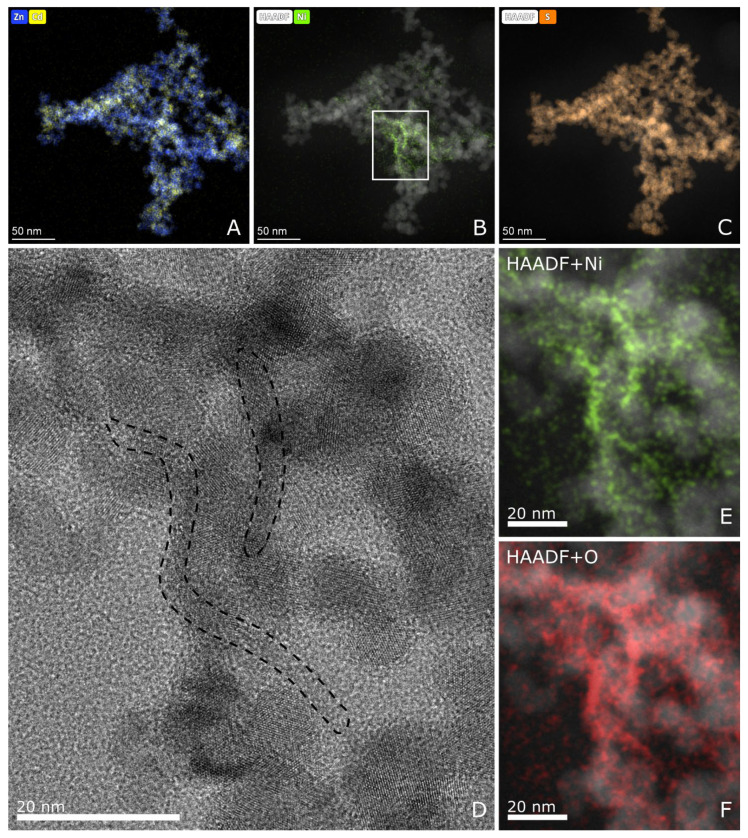
STEM composite images of Cat-4H created from the elemental maps and the HAADF signal (**A**–**C**,**E**,**F**). The HRTEM image (**D**) and the elemental maps (**E**,**F**) showing the same area marked with the white rectangle on image (**B**). The area outlined by the black dashed line on (**D**) shows the Ni-rich zones. Figure S5 in the Supplementary Materials shows similar STEM images of Cat-4E.

STEM composite images created from the elemental maps and HAADF signal show the inhomogeneity of the Cd-Zn distribution (Figure 6A) and visualize the Ni-rich area (Figure 6B). According to the S (Figure 6C), O (Figure 6F), and the Ni (Figure 6B,E) maps, the Ni-phase is clearly separated from the bulk of CdS-ZnS nanocrystals and could mostly be observed between or on the surface of CdS-ZnS crystallites independently on the type of Ni(II) modification (Figure S5 in the Supplementary Materials). From the elemental maps, it is striking that the Ni-rich areas are poor in S but rich in O, which clearly indicates that Ni^2+^, despite being precipitated by the addition of Na_2_S, forms nickel(II) hydroxide and/or nickel(II) oxide (NiO_x_) instead of NiS. The crystal structure of the Ni-rich phase could not be identified because of the small crystal size and/or poorly ordered structure.

The results of the TEM analysis also confirm that the Ni modification, regardless of the method used, does not affect the crystal structure of CdS-ZnS in a noticeable way. Similar results were obtained earlier for Ni modification of CdS or CdS_0_._4_ZnS_0_._6_ catalysts, which were prepared by methods deviating from our procedures [37,50]. The DRS, XRD, and TEM analyses together confirm that the Ni^2+^ ion is not embedded into the lattice structure of CdS-ZnS, so its effect on modifying the efficiency can only occur by surface interactions. Two possible roles of NiO_x_ should be considered. On the one hand, by acting as a co-catalyst, electron transfer between NiO_x_ and Cd_0_._25_Zn_0_._75_S catalysts can occur. On the other hand, by partially coating the surface of the CdS-ZnS composite, it can absorb a part of the incident photons.

**Figure 7:**
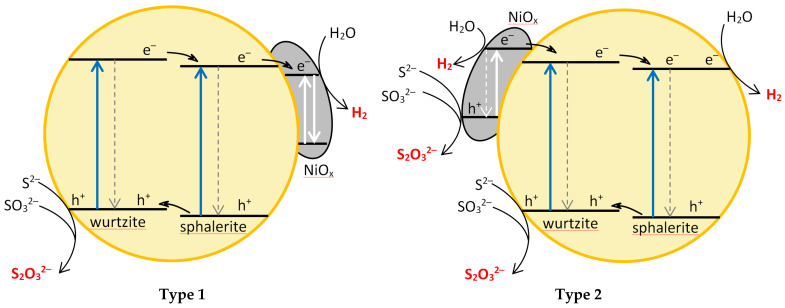
Schematic illustration of the proposed role of NiOx co-catalyst for enhancing the RHP.

Depending on the CB potential, NiO_x_ can play two different roles (Figure 7). If the CB electrons of NiO_x_ are at a lower energy level than those of the CdS-ZnS catalyst (Type 1 in Figure 7), NiO_x_ traps the CB electrons of Cd_0_._25_Zn_0_._75_S, reducing the rate of recombination. If the CB potential of the co-catalyst is more negative than that required for H_2_ generation, the catalytic efficiency is increased. Since, in this case, the direct light absorption of NiO_x_ does not cause efficient electron–hole separation, if the Ni(II) compound covers too high a fraction of the catalyst surface, the ratio of the photons absorbed by the CdS-ZnS catalyst will diminish, leading to a decrease in catalytic activity.

Another possibility would be if the CB potential of NiO_x_ is more negative than that of Cd_0_._25_Zn_0_._75_S (case type 2 in Figure 7). In this case, NiO_x_ can transfer electrons to the CdS-ZnS composite after photoexcitation, and the catalyst–co-catalyst roles would be swapped compared to the “Type 1” case. If this scenario were to occur, the higher the Ni-content, the greater the fraction of incident photons would be absorbed. This would lead to a monotonous increase in efficiency with Ni-content. Since the enhancement of the Ni-content only resulted in an increase in activity of up to 0.1–0.2% for the Ni-modified catalysts investigated, we propose the type 1 mechanism. This was also confirmed by the experience that for heat-treated catalysts with a lower specific surface area, the maximum catalytic activity was observed at lower Ni contents.

### 2.3. Stability of Catalysts

**Figure 8:**
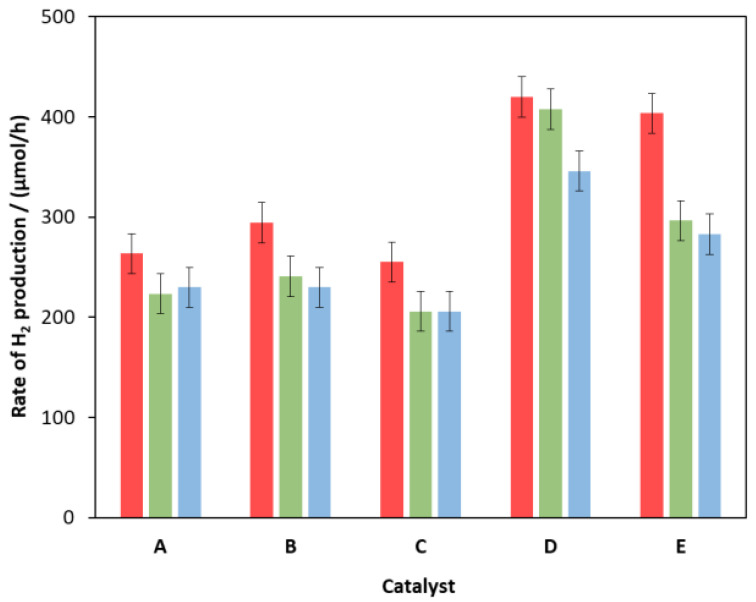
Changes in RHP over 3 consecutive illuminations for some catalysts: hydrothermally treated unmodified (**A**); hydrothermally treated and modified with 0.1% Ni(II) in the bulk (**B**) or on the surface (**C**); hydrothermally untreated and modified with 0.1% (**D**) and 0.3% (**E**) Ni(II) on the surface.

The photostability of some catalysts was investigated by three consecutive illuminations (Figure 8). Between the illumination experiments, the catalyst was separated by centrifugation, and the solution of the sacrificial electron donor was replaced with a fresh one.

After the first 24-hour irradiation, a 10–20% decrease was observed for most catalysts, but after that, the RHP was stabilized, which is most obvious from the time dependence of the RHP as shown in the Supplementary Materials (Figure S6 in the Supplementary Materials). The highest stability was measured for the hydrothermally untreated composite prepared from a solution containing an excess of ammonia and then modified with 0.1% NiS on the surface (Figure 8D). Although the initial activity of the similarly prepared composite containing 0.3% NiS (Figure 8E) was higher, its stability decreased by more than 30% after the first use, confirming the optimality of sample D (Figure 8).

**Figure 9:**
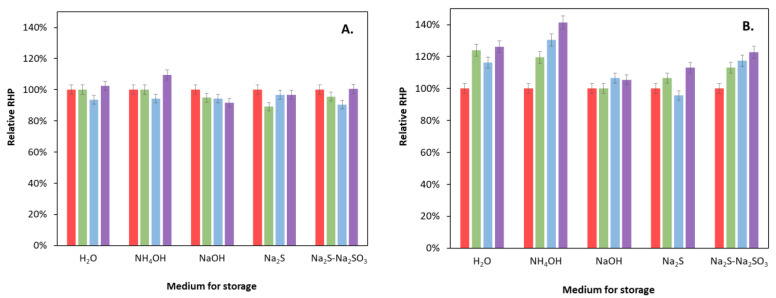
Changes in the *RHP* for hydrothermally treated catalysts (unmodified (**A**); modified with 0.5% Ni(II) in the bulk (**B**)) during storage in various media. Red, green, blue, and violet colors indicate the results after 0, 4, 7, and 12 months of storage, respectively. (The red columns, indicating the starting states, represent the references, i.e., 100%).

Since we did not dry the prepared catalysts after washing (as most research groups usually do) but stored them in aqueous suspension, the changes in the activity during 4-, 7-, and 12-month periods of storage in different media were also investigated for an unmodified (Cat-2NH) and a 0.5% NiS bulk-modified catalyst (Cat-2NH-0.5%Ni-B) (Figure 9). Another aim of this study was to investigate whether different storing media have an effect on the catalyst’s efficiency. Milli-Q water, 2 M NH_4_OH, 2 M NaOH, 0.1 M Na_2_S, and 0.1 M Na_2_S + 0.1 M Na_2_S_2_O_3_ solutions were used as testing media. In the case of the Cat-2NH catalyst, no change in RHP beyond the measurement error was observed in any of the media tested, even after one year of storage. Only a very slight decrease in the efficiency could be observed after storing in NaOH solution. In contrast, the Ni(II)-modified composite showed a slight increase in all cases, which was most significant when it was stored in an ammonia solution. This can probably be explained by the fact that the solubility of NiS or Ni(OH)_2_ in ammonia solution is better than in the other tested media due to the formation of an ammine complex, which favors slow recrystallization.

## 3. Materials and Methods

### 3.1. Materials

Zinc and cadmium acetate dihydrate (Zn(CH_3_COO)_2_⋅2H_2_O, Cd(CH_3_COO)_2_⋅2H_2_O), nickel(II) nitrate hexahydrate (Ni(NO_3_)_2_⋅6H_2_O), hexachloroplatinic acid hydrate (H_2_[PtCl_6_]⋅H_2_O), and sodium thiosulfate pentahydrate (Na_2_S_2_O_3_⋅5H_2_O) were purchased from Reanal (Budapest, Hungary) and sodium sulfide nonahydrate >98% (Na_2_S⋅9H_2_O) from Acros Organics (Geel, Belgium). The water applied was cleaned by a Millipure Elix equipment (Millipore S.A.S., Molsheim, France) completed with a Milli-Q 50 purification system (Millipore S.A.S., Molsheim, France). The solutions containing sulfide/sulfite were prepared in advance by using argon bubbled Milli-Q water and kept in the freezer for further experiments.

### 3.2. Photocatalyst Preparation

The preparation of unmodified ZnS-CdS catalyst was published in our earlier work [42]. Briefly, 1 mmol cadmium acetate dihydrate and 3 mmol zinc acetate dihydrate were dissolved in 10 mL Milli-Q water, 0, 1.5 (stoichiometric amount), or 3.0 mL of 25% ammonia solution was added, and the mixture was stirred for 5 min. Then, 4.4 mmol (10% excess) of Na_2_S⋅9H_2_O was dissolved in 10 mL Milli-Q water, which was deaerated by bubbling of argon gas for 20 min. This Na_2_S solution was added to the solution containing the metal ions under vigorous stirring. The suspension was divided into two parts. One of them was stirred for another 15 min, while the other part was poured into a 50 mL Teflon-lined autoclave and hydrothermally treated at 170 °C for 3 h. Both parts were yellowish precipitates that were washed twice with Milli-Q water and centrifuged. The catalysts were then stored in water suspension. The compositions of the unmodified catalysts were checked in our previous work [42] by EDS analyses, and the measured Zn:Cd ratio proved to be 3.0 ± 0.1.

These catalysts were modified with Ni^2+^ in two different ways. Bulk modified catalysts (“Ni-B”) were obtained by the addition of appropriate volumes of 0.1 M nickel(II) nitrate to the initial solution containing the acetate salts before the sulfide precipitation. In all of these cases, 3.0 mL of 25% ammonia solution was added, and hydrothermal treatment was applied as described above.

The surface-modified catalysts (“Ni-S”) were prepared from the hydrothermally treated and untreated unmodified catalysts by the addition of suitable volumes of 0.1 M nickel (II) nitrate. After 10 min stirring, 20% excess sodium sulfide was added, and the suspension was stirred for another 10 min. The freshly obtained composite was used for photochemical experiments. The Ni content was always given in relation to the total amount of metals (*x*_Ni_ = *n*_Ni_ / (*n*_Cd_ + *n*_Zn_ + *n*_Ni_). Similarly, for the purpose of comparison, Pt was applied on the surface of the unmodified catalyst by the addition of a suitable amount of 0.1 M hexachloroplatinic acid solution instead of 0.1 M Ni(NO_3_)_2_. In this case, irradiation was applied for the deposition of Pt; it was finished within the first 8 h of illumination (under the circumstances used for hydrogen generation). Notably, in the case of Pt-modified catalysts, the rate of H_2_ production was measured by the second irradiation cycle. The nickel contents were checked by ICP measurements for the photocatalyst samples, which proved to be the most efficient. The results of these determinations indicated good agreements between the theoretical and experimental values (for Ni, 0.10 mol% vs. 0.11 ± 0.01 mol% and 0.5 mol% vs. 0.44 ± 0.01 mol%, the latter data regard a less efficient sample). In the case of platinum, the method is considerably less sensitive than for Ni. Even so, 0.07 ± 0.04% could be detected.

### 3.3. Characterization

The crystallite size and phase composition were determined by X-ray diffraction measurement (Philips PW3710, Cu Kα radiation, 50 kV, and 40 mA). Data collections were carried out with an X’Pert Data Collector software (2.0e, PANalytical B.V., Almelo, the Netherlands, 2010). The full width at half-maximum (FWHM) values of the individual reflections was determined by the profile fitting treatment of the HighScore Plus software (5.0, Malvern Panalytical B.V., Almelo, the Netherlands, 2021). The peak broadening caused by the samples was explained by the presence of very small crystallites. The broadening of the 111 reflections of hawleyite and sphalerite (measured breadth minus the instrumental breadth) was used to calculate the average crystallite size by the well-established Scherrer equation [53]. The FWHM of the 100 reflections of the ZnO (heated at 1000 °C) was employed as the instrumental breadth. The 01-075-0581, 00-005-0566, 00-006-0314, and 00-036-1450 Powder Diffraction File (PDF) of ICDD (International Centre for Diffraction Data) of hawleyite, sphalerite, greenockite, and wurtzite, respectively, were used to identify phases.

Specord S600 spectrofluorometer equipped with an integrating sphere (Analytic Jena GmbH, Jena, Germany) was used to measure the diffuse reflectance spectra and deduce the BG with the Kubelka–Munk function.

Samples for transmission electron microscopy (TEM) were prepared by depositing a drop of diluted aqueous suspension of the original samples on copper TEM grids covered by continuous carbon amorphous support film. TEM analyses were performed using a Talos F200X G2 instrument (Thermo Fisher Scientific, The Netherlands), operated at 200 kV accelerating voltage, equipped with a field-emission gun and a four-detector Super-X energy-dispersive X-ray spectrometer (Termo Fisher Scientific), and capable of working in both conventional TEM and scanning transmission (STEM) modes. In our study, TEM bright-field images, HRTEM images, and STEM high-angle annular dark-field (HAADF) images were collected to visualize the crystal size, and the morphology of the particles, and HRTEM images as well as electron diffraction patterns were used to study the structural properties, and STEM-EDS elemental maps were collected to measure and visualize the chemical compositions. Elemental analysis (Ni, Pt) was carried out by means of Spectroflame Module E type (SPECTRO Analytical Instruments GmbH, Kleve, Germany) ICP-OES instrument. Samples were nebulized into 4.6 argon plasma using a horizontal torch and axial plasma viewing. Applied emission lines were 265.945 nm and 231.604 nm for Pt and Ni, respectively.

### 3.4. Photochemical Experiments

The photochemical experiments were performed under the conditions described by Fodor et al. [27] in a double-necked reactor of 40 cm^3^ volume (14 mm height, 60 mm diameter). The total volume of all sacrificial solutions was 30 cm^3^, and they contained 0.117 M Na_2_S, 0.16 M Na_2_SO_3_, and 18 mg of catalyst (0.6 g/dm^3^). Before illumination, all samples were deaerated by argon bubbling for 10 min. Two 7 W 6000 K Optonica visible LEDs (Optonica LED, Sofia, Bulgaria) were used as light source. For the determination of the quantum yield, a 380-nm LED with 13 nm full width at half maximum (fwmh) was used. Its intensity was determined by a trioxalato-ferrate(III) actinometer. The amount of the photon incident in the reactor was found to be 7.31 μmol/s (light intensity: 83.9 mW/cm^2^). The emission spectra of these light sources are depicted in the Supplementary Materials (Figure S7).

The evolved hydrogen was bubbled into a vessel filled with 1 mM NaOH solution, and its volume was calculated from the mass of the NaOH solution displaced. Mass data were collected every minute by using a Kern PCB balance (Kern & Sohn GmbH, Balingen, Germany) connected to a PC. The illuminations were always performed until the end of reactions. The amount of evolved hydrogen was equal to the initial amount of Na_2_S in all cases. The validity of the method applied was proved in our previous paper [27]. A similar water-displacing method was used by Zhang et al. to measure the volume of hydrogen generated [54]. Before starting each irradiation, the system was checked regarding gas leakage. The RHP values were calculated as the average ones for the 200–400 min interval. For many, but not all catalysts, parallel illuminations of at least three independent portions of a catalyst sample were performed. Based on this experience, the errors of the plotted average RHP values were estimated to be ~0.5 mL/h (i.e., 20 μmol/h). Additionally, error bars have also been inserted into the corresponding diagrams. The experiments were carried out at room temperature; the photoreactor was not thermostated. However, the warming effect of the irradiations was measured as described in the Supplementary Materials (see Figure S8 and the corresponding text below that). The temperature of the irradiated reaction mixtures was 42–48 °C at the end of the illumination. More importantly, the reaction mixture was not stirred during the irradiation to approach the conditions of practical (industrial) applications. Nevertheless, some experiments were carried out with stirring, too, and the cumulative hydrogen production was about the same as without mixing.

## 4. Conclusions

For catalysts not modified with Ni, HTT clearly and significantly increased the RHP regardless of the amount of NH_3_ applied during precipitation. However, modification of hydrothermally treated catalysts with Ni(II) caused, at most, a 10–20% increase in the catalytic efficiency at low Ni contents. In contrast, surface modification of the hydrothermally untreated catalyst with 0.1–0.3% Ni(II) resulted in more than a two-fold increase in the efficiency compared to the unmodified composite. This activity was 15% better than the best HT-treated sample and 20% better than the Pt-modified one. A 14.9% QY was achieved for this catalyst by illuminating with 380 nm light.

The structural characterization of the composites clearly confirms that Ni^2+^ ions, modifying either the bulk or the surface of the catalyst, are not embedded into the CdS-ZnS crystal lattice but are enriched on the surface of particles of the original catalyst in the form of NiO or Ni(OH)_2_ (NiO_x_). Based on these conclusions, the reasonable explanation of our photochemical results is that NiO_x_ acts as a co-catalyst and promotes the trapping of CB electrons, thus increasing the efficiency, but if it excessively covers the surface, it inhibits the excitation of the semiconductor particles and, thus, prevents the formation of the photogenerated electron-hole pairs. The main advantages of our most efficient catalyst are that it does not require energy-intensive hydrothermal treatment to produce, and its very low Ni(II) content does not increase the cost of production.

## Figures and Tables

**Table 1:** The band-gap energies of different catalysts (precipitated from solution of ammonia in 2-fold excess) determined from Tauc-representation [52] of KM-functions (Figure S4 in the Supplementary Materials). The “Ni-B” and “Ni-S” designations (in parentheses) represent the bulk and the surface modifications, respectively.

	Band-Gap Energy/eV	
Ni Content (%)	HT Treated (Ni-B)	HT Untreated (Ni-S)
0	2.59 ± 0.02	2.68 ± 0.03
0.1	2.59 ± 0.02	2.73 ± 0.03
0.2	2.58 ± 0.02	2.72 ± 0.03
0.3		2.70 ± 0.03
0.4		2.70 ± 0.03
0.5	2.57 ± 0.02	2.65 ± 0.03
1.0	2.57 ± 0.02	
2.0	2.61 ± 0.02 *	

* This value has been estimated by base-line correction (see Figure S8B) because the formation of NiS increased the background absorption, although it does not affect the band gap, i.e., its excitation does not lead to efficient generation of electron–hole pairs [38].

## Data Availability

The data presented in this study are available on request from the corresponding author. The data are not publicly available due to privacy.

## Supplementary Materials

The following are available online at https://www.mdpi.com/article/10.3390/molecules27134296/s1, Figure S1: The volume of evolved H_2_ (A,C,E) and the rate of H_2_ evolution (B,D,F) for catalysts prepared from 2-fold excess of ammonia and modified with different amounts of Ni(II) in the bulk (A,B) and on the surface (C–F). The catalysts in (A–D) were hydrothermally treated, while the catalysts in (E,F) were not treated. The functions in (B, D, and F) are the time derivatives of those in (A, C, and E), respectively; Figure S2: The volume of evolved H_2_ (A) and the rate of H_2_ evolution (B) for unmodified catalysts (yellow and orange squares) and for catalysts modified with 0.1% Pt (blue triangles) and 0.1% Ni(II) (green and gray circles) on the surface. “2NH” (filled symbols) and “2N0H” (open symbols) represent the hydrothermally treated and untreated catalysts, respectively. The functions in (B) are the time derivatives of those in (A); Figure S3: The volume of evolved H_2_ for catalysts precipitated from solutions containing different amounts of ammonia ((A): no NH_3_, (B): stoichiometric amount of NH_3_, (C): 2-fold excess, and (D): 4-fold excess of NH_3_ were applied). Yellow and blue symbols represent the hydrothermally untreated (“2N0H”), while orange and gray symbols represent the HT treated (“2NH”) catalysts. The blue and gray curves symbolize the surface-modified catalysts with 0.1% Ni content (“01NiS”), while the yellow and orange ones belong to the unmodified catalysts (“0NiS”)., Figure S4: The Tauc representation of hydrothermally untreated (A) and treated (B) catalysts modified with various amounts of Ni(II) on the surface (A) or in the bulk (B)., Figure S5. HRTEM image and STEM elemental maps of Cat-4E catalyst., Figure S6: Changes in RHP over 3 consecutive illuminations for hydrothermally treated catalyst modified with 0.1% Ni(II) in the bulk (A) and for hydrothermally untreated catalyst modified with 0.3% Ni(II) on the surface (B). Red, green, and blue circles represent the RHP for the 1st, 2nd, and 3rd illuminations, respectively. Figure S7: Normalized intensity of the light sources applied for illuminations (blue line: 380 nm UV-LED, green line: vis-LED), and the KM-function of HTT, unmodified catalyst., Figure S8: Results of a blank irradiation experiment. The volume of a displaced solution (blue) and the temperature of the reaction mixture (red) containing 30 mL 0.117 M Na_2_S, 0.160 M Na_2_SO_3_ and 18 mg of CdS.
