# Supplementary material for: Effects of Preparation Conditions on the Efficiency of Visible-Light-Driven Hydrogen Generation Based on Ni(II)-Modified Cd0.25Zn0.75S Photocatalysts
Source: Molecules. 2022 Jul 4;27(13):4296. doi: 10.3390/molecules27134296 (PMC9268298; doi:10.3390/molecules27134296)
Supplement: Supplementary file 1 [file molecules-27-04296-s001.zip › molecules-1774440-supplementary.pdf]

Effects of preparation conditions on the efficiency of visible-light-driven hydrogen generation based on Ni(II)-modified $Cd_{0.25}Zn_{0.75}S$ photocatalysts

Maali-Amel Mersel[1], Lajos Fodor[1], Péter Pekker[2], Éva Makó[3], and Ottó Horváth[1,*]

[1] Research Group of Environmental and Inorganic Photochemistry, Center for Natural Sciences, Faculty of Engineering, University of Pannonia, P.O.B. 1158, Veszprém H-8210, Hungary; sam003miloo@gmail.com (M-A.M.); lajos@almos.uni-pannon.hu (L.F.)

[2] Environmental Mineralogy Research Group, Research Institute of Biomolecular and Chemical Engineering, University of Pannonia, H-8210 Veszprem, POB. 1158, Hungary; pekkerpeter@gmail.com (P.P.)

[3] Department of Materials Engineering, Research Center for Engineering Sciences, University of Pannonia, H-8210 Veszprem, POB. 1158, Hungary; makoe@almos.uni-pannon.hu (É. M.)

* Correspondence: horvath.otto@mk.uni-pannon.hu (O.H.); Tel+36-88-624-000 / 6049 ext.

Content **Page Nr.**

Figure S1 2

Figure S2 3

Figure S3 4

Figures S4,5 5

Figures S6,7 6

Figure S8 with text 7

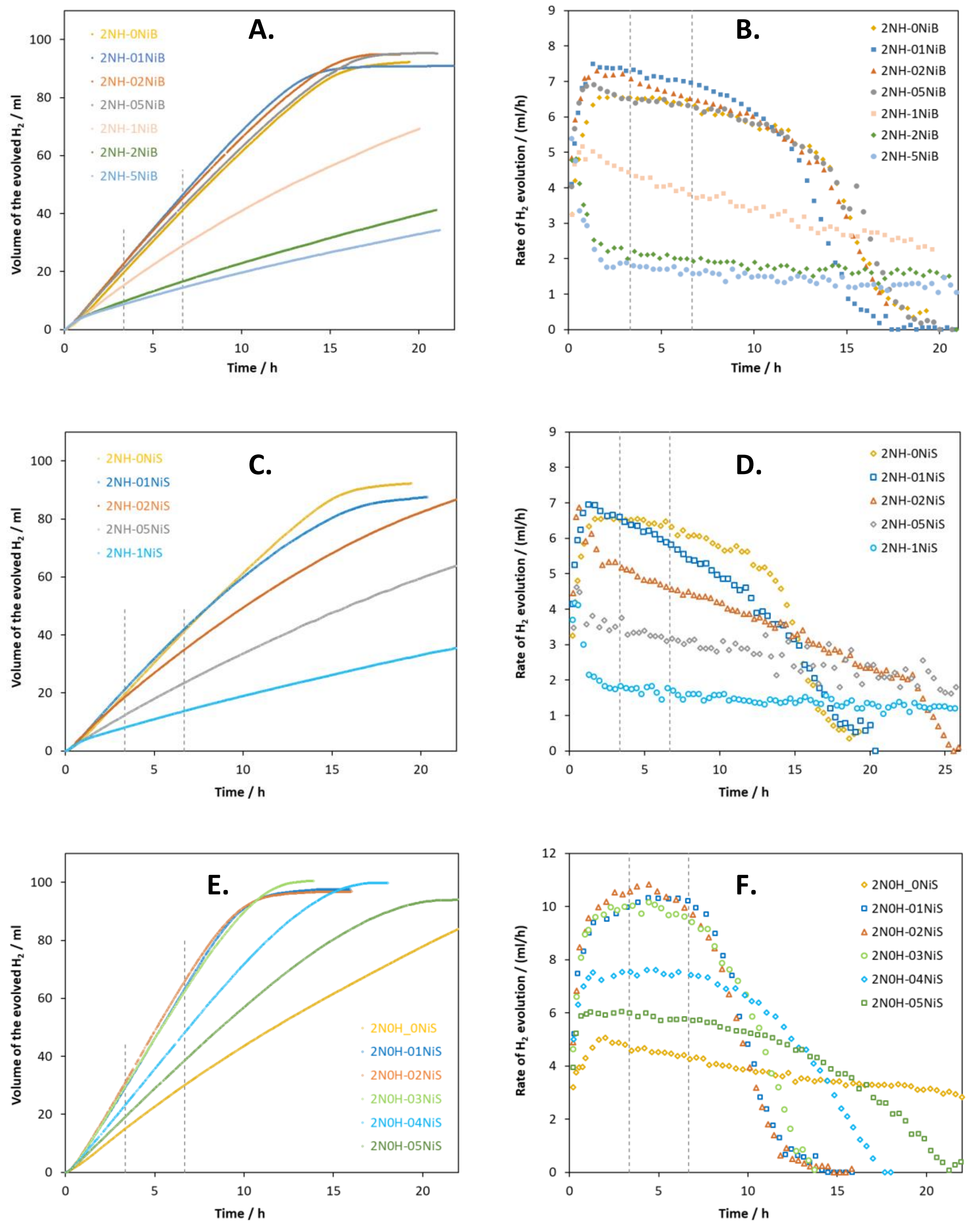

Figure S1. The volume of evolved H_2 (A, C, E) and the rate of H_2 evolution (B, D, F) as functions of time for catalysts prepared from 2-fold excess of ammonia and modified with different amounts of Ni(II) in the bulk (A, B) and on the surface (C-F). The catalysts in (A-D) were hydrothermally treated, while the catalysts in (E-F) were not treated. The functions in (B, D, and F) are the time derivatives of those in (A, C, and E), respectively.

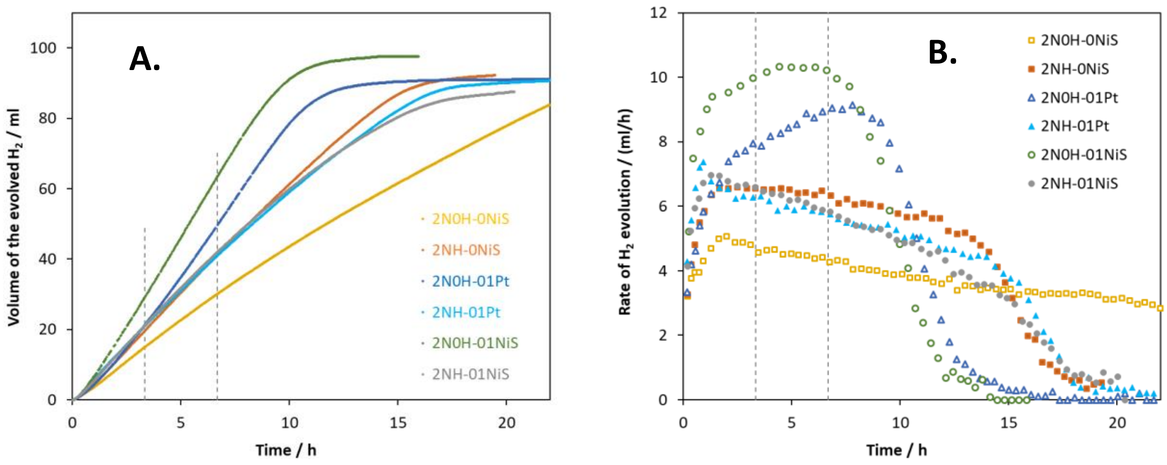

Figure S2. The volume of evolved H_2 (A) and the rate of H_2 evolution (B) as functions of time for unmodified catalysts (yellow and orange squares) and for catalysts modified with 0.1% Pt (blue triangles) and 0.1 % Ni(II) (green and gray circles) on the surface. "2NH" (filled symbols) and "2N0H" (open symbols) represent the hydrothermally treated and untreated catalysts, respectively. The functions in (B) are the time derivatives of those in (A).

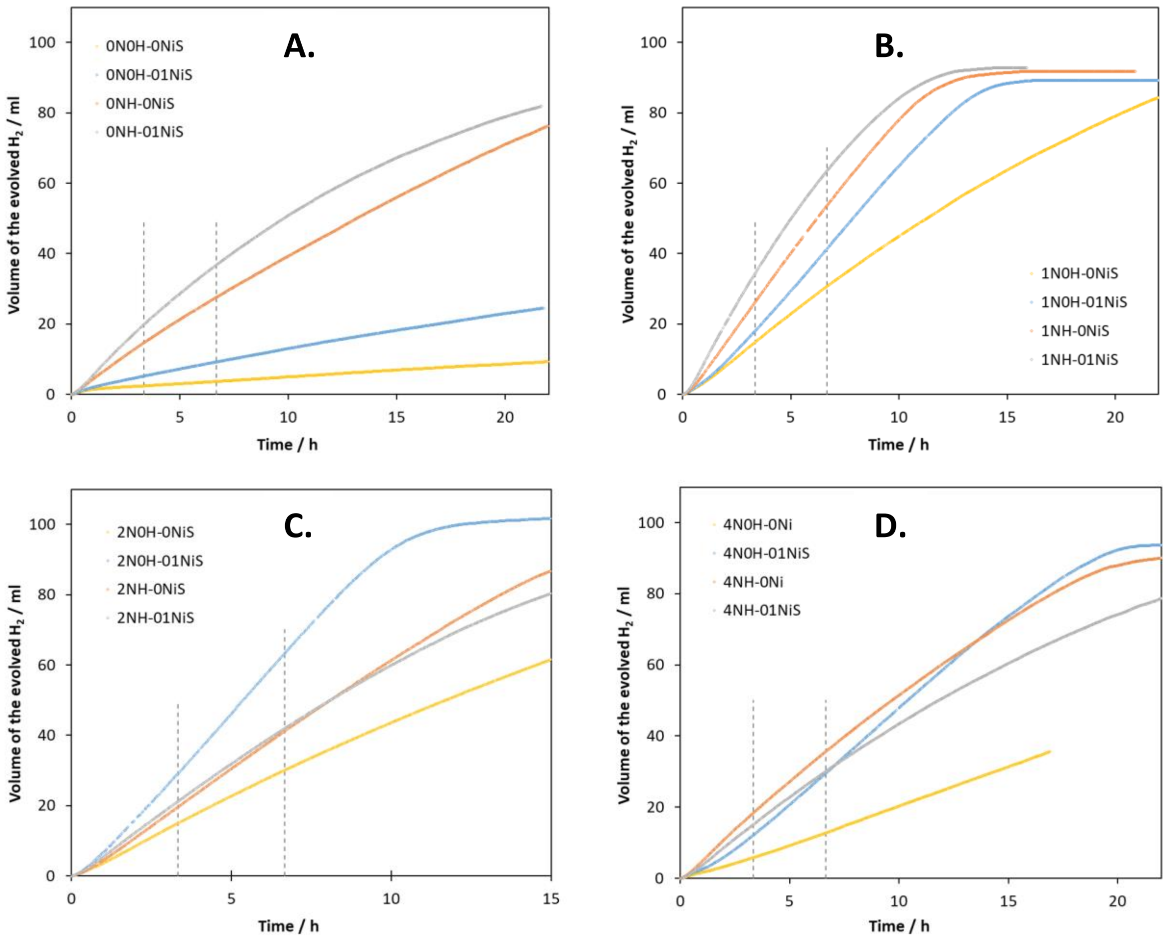

Figure S3. The volume of evolved H_2 as functions of time for catalysts precipitated from solutions containing different amounts of ammonia ((A): no NH_3, (B): stoichiometric amount of NH_3, (C): 2-fold excess, and (D): 4-fold excess of NH_3 were applied). Yellow and blue symbols represent the hydrothermally untreated ("2N0H"), while orange and gray symbols represent the HT treated ("2NH") catalysts. The blue and gray curves symbolize the surface-modified catalysts with 0.1% Ni content ("01NiS"), while the yellow and orange ones belong to the unmodified catalysts ("0NiS").

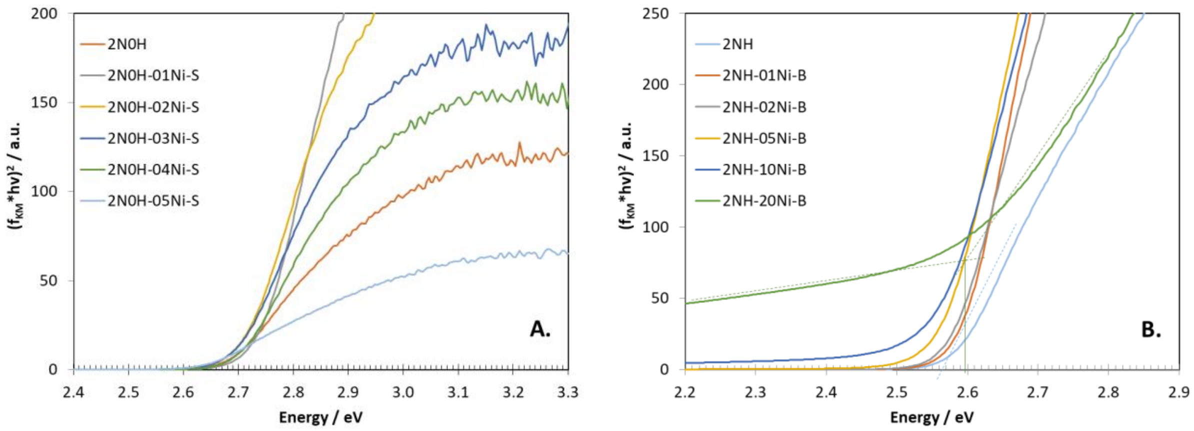

Figure S4. The Tauc representation of hydrothermally untreated (A) and treated (B) catalysts modified with various amounts of Ni(II) on the surface (A) or in the bulk (B).

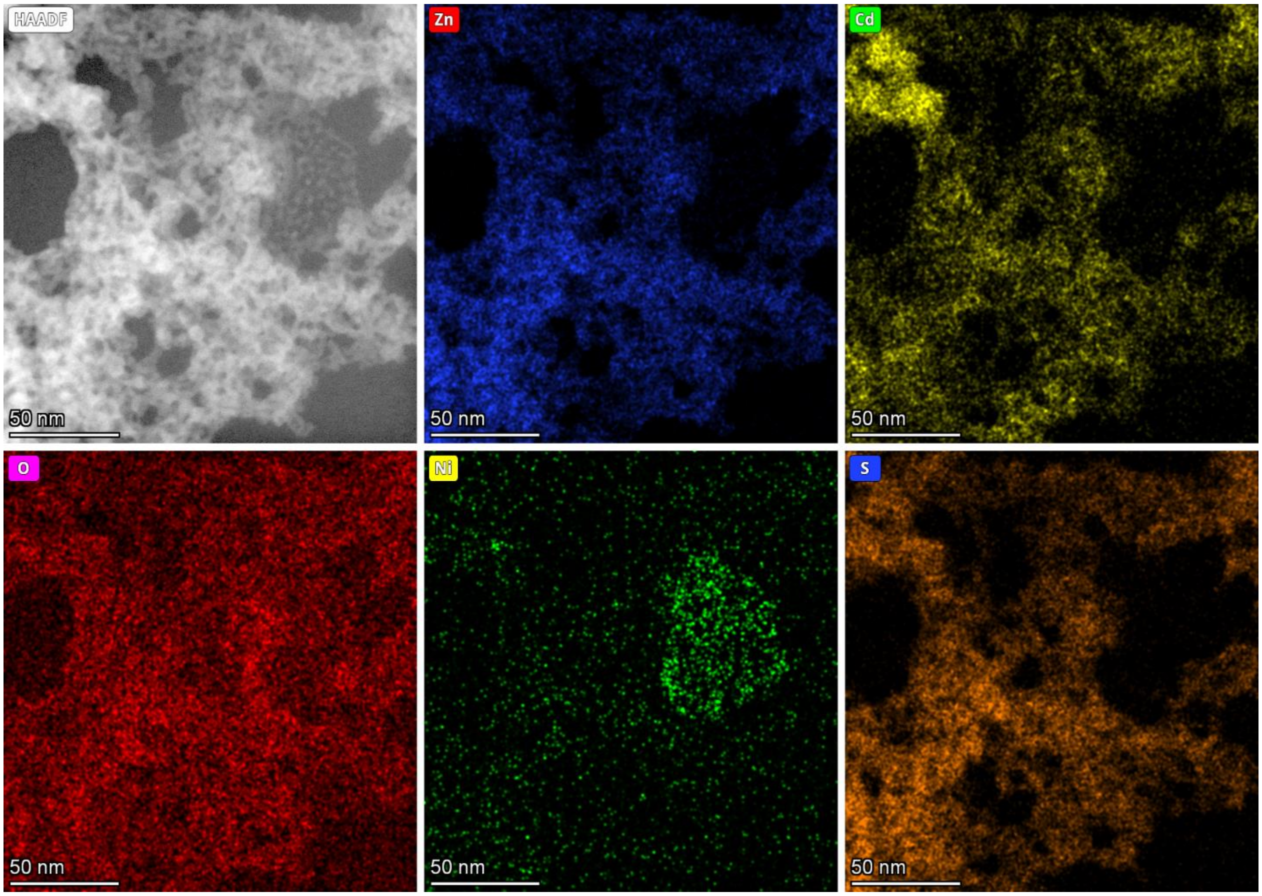

Figure S5. HRTEM image and STEM elemental maps of Cat-4E catalyst.

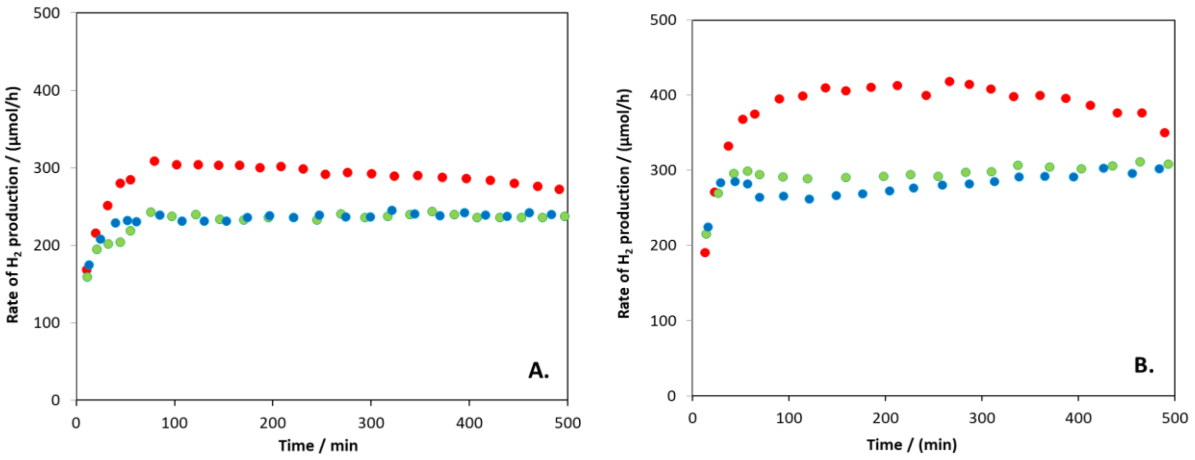

Figure S6. Changes in RHP over 3 consecutive illuminations for hydrothermally treated catalyst modified with 0.1% Ni(II) in the bulk (A) and for hydrothermally untreated catalyst modified with 0.3% Ni(II) on the surface (B). Red, green, and blue circles represent the RHP for the 1st, 2nd, and 3rd illuminations, respectively.

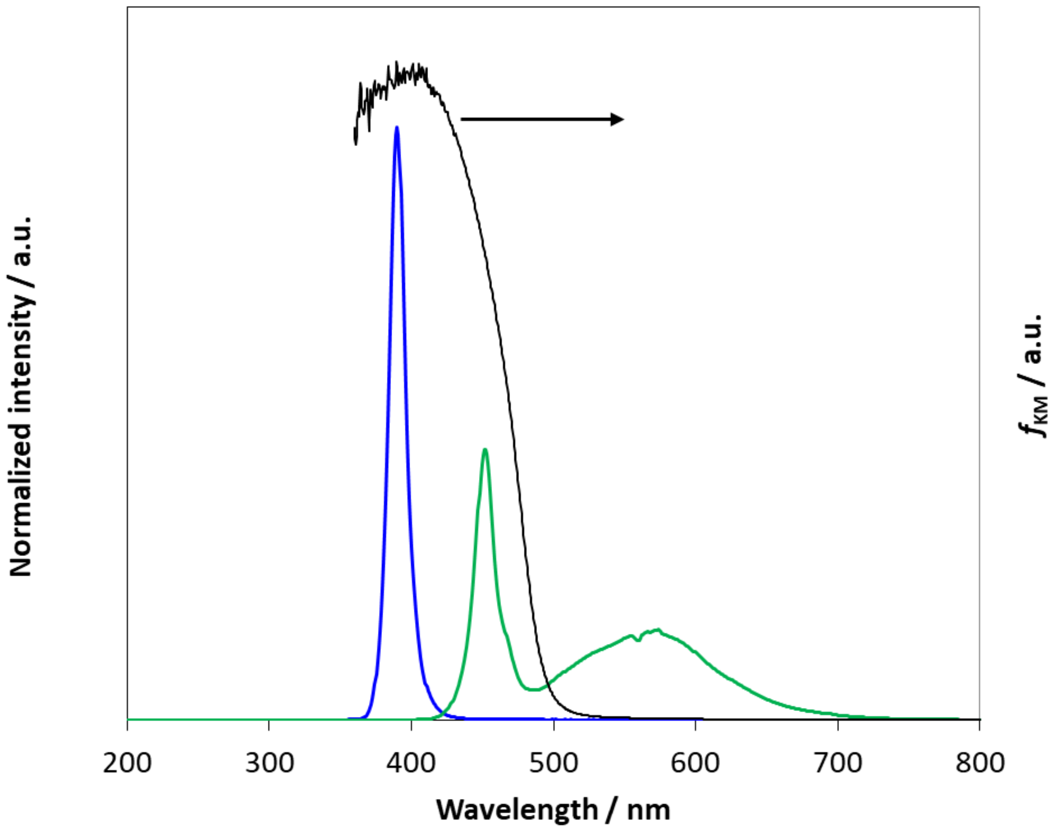

Figure S7. Normalized intensity of the light sources applied for illuminations (blue line: 380 nm UV-LED, green line: vis-LED), and the KM-function of HTT, unmodified catalyst.

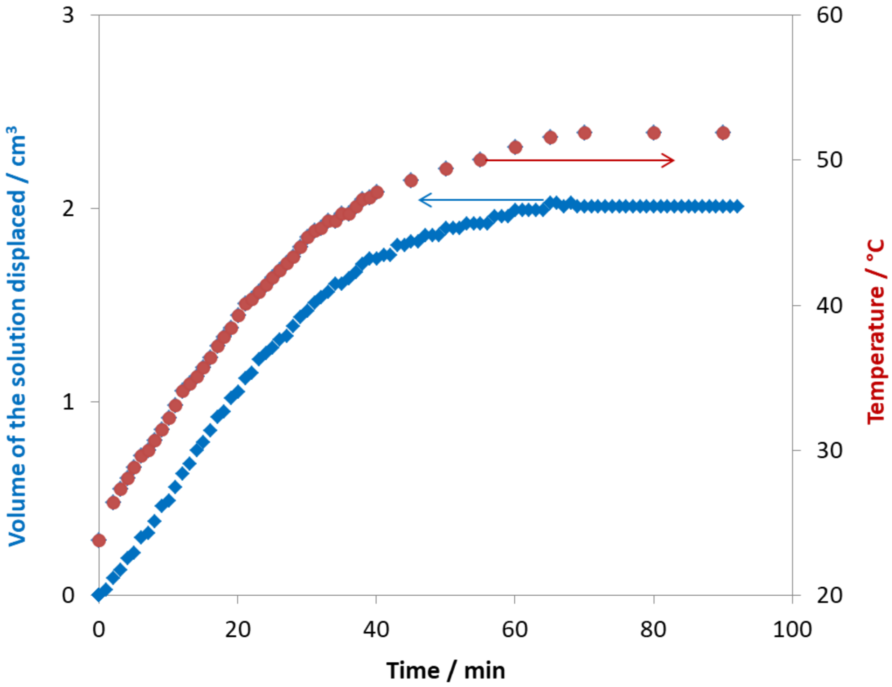

Figure S8. Results of a blank irradiation experiment. The volume of a displaced solution (blue) and the temperature of the reaction mixture (red) containing 30 ml 0.117 M Na_2S, 0.160 M Na_2SO_3 and 18 mg of CdS.

Since the fraction of photons absorbed by pure CdS is higher than for any catalyst we applied, the change of the temperature in this experiment is higher than it could be in any other case. The temperature of the reaction mixture at the end of the irradiation was typically between 42 °C and 48 °C. The thermal equilibrium was achieved within about 1 hour, during this period the volume of the displaced liquid due to thermal expansion was 1.5-2.0 cm^3.
